# Nanowire-Enhanced Fully Transparent and Flexible Indium Gallium Zinc Oxide Transistors with Chitosan Hydrogel Gate Dielectric: A Pathway to Improved Synaptic Properties

**DOI:** 10.3390/gels9120931

**Published:** 2023-11-27

**Authors:** Dong-Hee Lee, Hamin Park, Won-Ju Cho

**Affiliations:** 1 Department of Electronic Materials Engineering, Kwangwoon University, Gwangun-ro 20, Nowon-gu, Seoul 01897, Republic of Korea; 2 Department of Electronic Engineering, Kwangwoon University, Gwangun-ro 20, Nowon-gu, Seoul 01897, Republic of Korea

**Keywords:** indium gallium zinc oxide, electric double layer, chitosan-based hydrogel electrolyte, random-network nanowire, polyvinylpyrrolidone template, synaptic transistor, artificial synapse, neuromorphic computing

## Abstract

In this study, a transparent and flexible synaptic transistor was fabricated based on a random-network nanowire (NW) channel made of indium gallium zinc oxide. This device employs a biocompatible chitosan-based hydrogel as an electrolytic gate dielectric. The NW structure, with its high surface-to-volume ratio, facilitated a more effective modulation of the channel conductance induced by protonic-ion polarization. A comparative analysis of the synaptic properties of NW- and film-type devices revealed the distinctive features of the NW-type configuration. In particular, the NW-type synaptic transistors exhibited a significantly larger hysteresis window under identical gate-bias conditions. Notably, these transistors demonstrated enhanced paired-pulse facilitation properties, synaptic weight modulation, and transition from short- to long-term memory. The NW-type devices displayed gradual potentiation and depression of the channel conductance and thus achieved a broader dynamic range, improved linearity, and reduced power consumption compared with their film-type counterparts. Remarkably, the NW-type synaptic transistors exhibited impressive recognition accuracy outcomes in Modified National Institute of Standards and Technology pattern-recognition simulations. This characteristic enhances the efficiency of practical artificial intelligence (AI) processes. Consequently, the proposed NW-type synaptic transistor is expected to emerge as a superior candidate for use in high-efficiency artificial neural network systems, thus making it a promising technology for next-generation AI semiconductor applications.

## 1. Introduction

In the context of recent strides in social platforms and artificial intelligence (AI), the volume of unstructured data processing, ranging from images and sounds to videos, has increased remarkably [1,2]. However, the conventional von Neumann computing architecture, rooted in sequential information processing, falters when confronted with this surge [3]. To surmount these inherent limitations, extensive research is being dedicated to synaptic devices that mirror the intricacies of the human brain [4,5,6,7,8]. Unlike conventional computing systems, the human brain seamlessly combines data storage and processing capabilities, enabling unparalleled parallel computing with remarkably low-power consumption [9]. 

To replicate these neural marvels, significant attention has been directed toward electric-double-layer (EDL)-based synaptic devices. These innovative constructs deploy solid electrolytes onto gate dielectrics, a strategy that is generating fervent interest [10,11,12]. Notably, proton-conducting chitosan electrolytes have emerged as standout solid electrolyte materials because of their affordability, biocompatibility, transparency, and flexibility [13,14]. Furthermore, the application of a gate bias polarizes the mobile protons within the chitosan matrix, thus facilitating the facile modulation of the channel conductance [15,16]. In turn, this conductance modulation engenders changes in synaptic plasticity, an elemental facet of the interconnection strength between pre- and post-synapses in the human brain [17]. 

Simultaneously, the potential applications of artificial synapses are rapidly expanding beyond data processing, encompassing domains such as soft robotics, artificial sensory nerves, neuroprosthetics, and wearable devices [18,19,20]. To fulfill these burgeoning possibilities, the development of flexible and transparent artificial synapses is imperative. However, despite numerous investigations into synaptic transistors utilizing diverse channels, such as graphene and pentacene, as well as various gate insulator materials, including AlO_x_ and albumen, studies focusing on harnessing structural adaptations to enhance synaptic properties remain limited [21,22,23,24]. In the quest for high-performance neuromorphic systems, the ramifications of structural alterations in synaptic properties warrant further in-depth exploration. Notably, the potential of nanowire (NW) channels to influence synaptic properties is promising. NW channels, which have a one-dimensional geometry and remarkable surface-to-volume ratio, have garnered significant interest across a spectrum of applications [25,26]. Their attributes, which range from high integration and precise charge control to reduced power consumption, render them ideal for artificial synaptic devices built on transistor configurations [27]. 

In this study, we propose a novel approach that implements a random-network NW channel structure to amplify the synaptic properties of flexible, transparent chitosan-based, hydrogel electrolyte, synaptic transistors. To enhance the synaptic properties through this NW channel architecture, we concurrently fabricated film-type devices utilizing identical materials. We employed electrospun polyvinylpyrrolidone (PVP) nanofibers as etching templates to fabricate In-Ga-Zn-O (IGZO) NWs with a randomized network arrangement. The ensuing experimental measurements of the electrical characteristics and hysteresis effects of NW-type synaptic transistors underpin the core of our investigation. Notably, we delved into essential synaptic functions, including the emulation of excitatory postsynaptic currents (EPSC), exploration of short- and long-term synaptic plasticity, and modulation of channel conductance. Moreover, our exploration is extended to artificial neural network (ANN) simulations by employing the Modified National Institute of Standards and Technology (MNIST) dataset for recognition experiments. 

Consequently, our NW-type device exhibits a performance that exceeds that of its film-type counterpart by showcasing a significantly expanded hysteretic window, a testament to its superior facilitation, long-term memory attributes, energy efficiency, and recognition accuracy. 

## 2. Results and Discussion

### 2.1. Electrical Characteristics of Transparent and Flexible IGZO NW Synaptic Transistors

**Figure 1:**
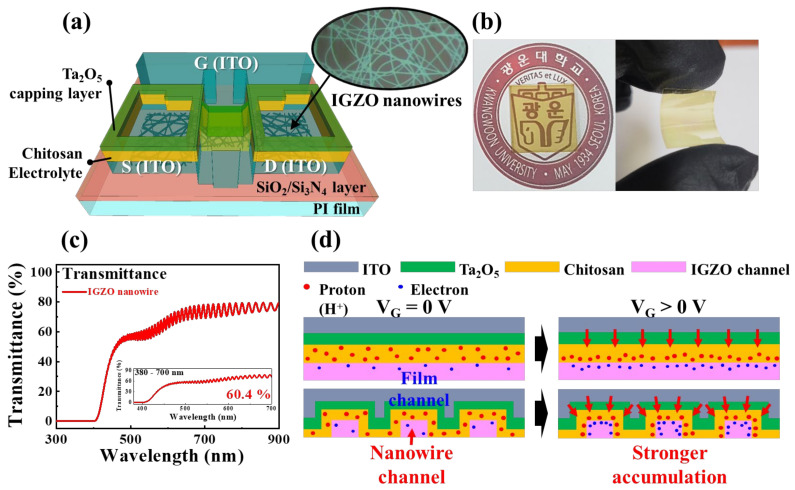
(**a**) Schematic and (**b**) photographic depiction of a fully transparent and flexible IGZO NW transistor integrated on a polyimide substrate. (**c**) Optical transmittance data (inset exhibits transmittance properties in the visible light region), (**d**) Illustration elucidating proton migration dynamics within the chitosan-based hydrogel electrolyte.

Figure 1a,b depict the transparent and flexible IGZO NW synaptic transistors, fabricated on PI substrates. Figure 1c represents optical transmittance data for NW-type EDLT fabricated on a flexible and transparent PI substrate. The inset exhibits data related to the visible light spectrum (380–700 nm). The average transmittance of the NW random networks was 60.4%, which was measured using an Agilent 8453 UV-visible spectrophotometer. Figure 1d shows a schematic of the modulation of synaptic plasticity induced by ionic polarization within a chitosan-based hydrogel electrolyte. This modulation was observed in synaptic transistors featuring both film- and NW-type channels. Protons constitute the predominant fraction of mobile ions within the chitosan polyelectrolyte matrix [28]. When a gate is subjected to a positive bias, these protons undergo polarization and migration, converging toward the interface shared by the channel and chitosan layers [29]. Of paramount significance is the retention of ionic polarization, even upon removal of the gate bias, thereby engendering a lingering influence on channel conductance that is instrumental in the instantiation of synaptic plasticity. Notably, owing to the distinctive attributes of the NW channel, such as its substantial surface-to-volume ratio and envelopment by the electrolyte, proton accumulation near the interface was amplified; this resulted in a heightened sway upon channel conductance. This distinctive characteristic invariably facilitated a more effective modulation of channel conductance under identical voltage conditions, culminating in an augmented manifestation of synaptic behavior. 

**Figure 2:**
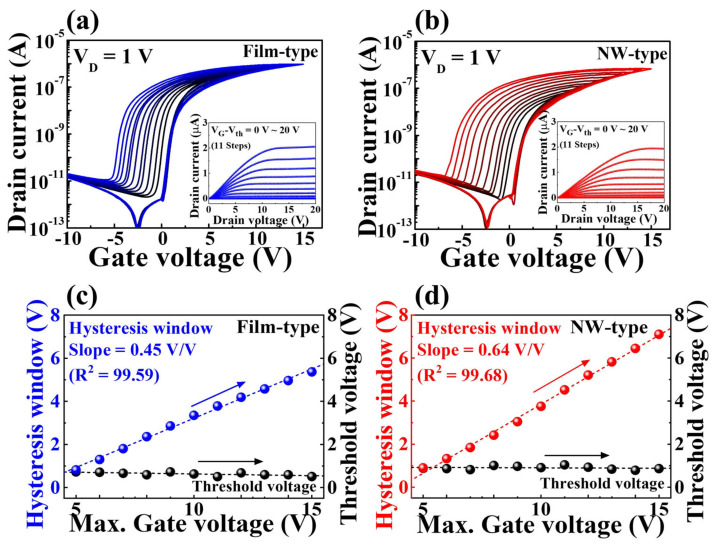
Double-sweep transfer curves (I_G_–V_G_) with increasing maximum V_G_ for (**a**) film-type and (**b**) NW-type synaptic transistors. Insets show corresponding output curves (I_D_–V_D_). Hysteresis window and threshold voltage for (**c**) film-type and (**d**) NW-type synaptic transistors.

Figure 2a,b show the double-sweep transfer curves, which show a progressive increase in the maximum gate voltage (V_G_). V_G_ was incrementally swept from −10 V to 5–15 V (1 V steps) and was then returned to −10 V, while maintaining a constant drain voltage (V_D_) of 1 V. Positive V_G_ values prompt the ionic polarization of mobile protons, thus resulting in a distinct counterclockwise hysteresis profile [30]. Notably, the elevation of the maximum V_G_ accentuates the ionic polarization, thereby broadening the hysteresis window. The hysteresis windows and threshold voltages extracted from the measured transfer curves are shown in Figure 2c,d, respectively. The hysteresis window is defined as the disparity in the V_G_ readings relative to the reference current (1 nA). Importantly, the hysteresis window demonstrated a linear augmentation in tandem at increasing maximum V_G_ values. Remarkably, the NW-type device surpasses its film-type counterpart in terms of the hysteresis enhancement rate, achieving a value of 0.64 V/V compared with 0.45 V/V (film-type device). This finding indicates a more effective modulation of channel conductance within the NW channel under equivalent conditions. In contrast, the threshold voltage remained stable; this was attributed to comprehensive ion depolarization facilitated by a substantial negative gate bias. The insets in Figure 2a,b provide insights into the output property curves (I_D_/V_D_) of the film- and NW-type devices. These properties were evaluated by sweeping V_D_ (from 0 to 20 V) within the |V_G_–V_TH_| range of 0 to 20 V. The insets show the output curves characterized by well-defined linear and saturated regions, signifying ohmic contact within the S/D electrodes and pinch-off phenomena [31]. Furthermore, the NW-type device demonstrated a lower I_D_ owing to its streamlined current pathway, which has the potential to mitigate power consumption in artificial synaptic devices. 

### 2.2. Synaptic Characteristics of Transparent and Flexible IGZO NW Synaptic Transistors

Paired-pulse facilitation (PPF), a short-term plasticity phenomenon observed in biological synapses, plays a crucial role in processing temporal information from visual or auditory signals [32,33]. This characteristic is fundamental to short-term synaptic plasticity and manifests itself as an amplification of the second excitatory postsynaptic current (EPSC) upon the application of consecutive pulse pairs, with its effect becoming more pronounced as the pulse time interval (Δt_interval_) decreases. The initial presynaptic spike leads to the accumulation of mobile protons at the electrolyte–channel interface. Subsequently, applying a second spike within a sufficiently short Δt_interval_ causes further accumulation of positive ions at the interface, increasing channel conductivity. 

**Figure 3:**
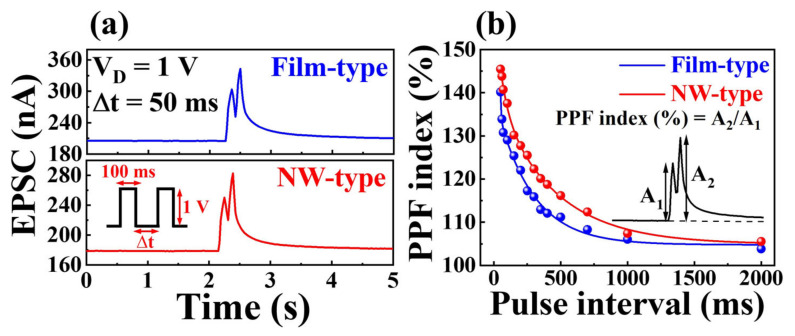
(**a**) Excitatory postsynaptic currents (EPSC) evoked by a paired-pulse (V_G_ = 1 V, 50 ms) with a pulse interval of 50 ms. (**b**) Paired-pulse facilitation (PPF) index plotted as a function of the pulse interval for the film- and NW-type channel synaptic transistors.

Figure 3a illustrates the EPSC (drain current at V_D_ = 1 V) triggered by a paired-pulse (V_G_ = 1 V, 50 ms) with an Δt_interval_ of 50 ms. Notably, the amplitude of the second EPSC (A_2_) surpasses that of the first EPSC (A_1_) because of the residual presence of protons near the channel following the first stimulus [34]. The behavior of PPF is characterized by the PPF index (A_2_/A_1_) plotted against Δt, as depicted in Figure 3b. The PPF index increases with decreasing Δt, and both device configurations effectively mimic the PPF behavior. While maintaining a fixed pulse duration of 50 ms and an amplitude of 1 V, the intervals were determined by incrementally increasing Δt from 50 ms to 2000 ms. The PPF index of the NW-type device reached values up to 145%, outperforming the film-type device’s value of 140% across the same Δt range. The derived PPF index was fitted using the double-exponential decay equation [35],

(1) $$PPF\;index=A+C_{1}exp(-\Delta t/\tau_{1})+C_{2}exp(-\Delta t/\tau_{2})$$

where A represents a constant value, C_1_ and C_2_ denote the initial facilitation magnitudes, and τ_1_ and τ_2_ correspond to characteristic relaxation times. These outcomes closely emulate biological synapses, underscoring the capacity of the proposed device to replicate the synaptic timescale and capture both slow and quick stages spanning tens to hundreds of milliseconds. 

**Figure 4:**
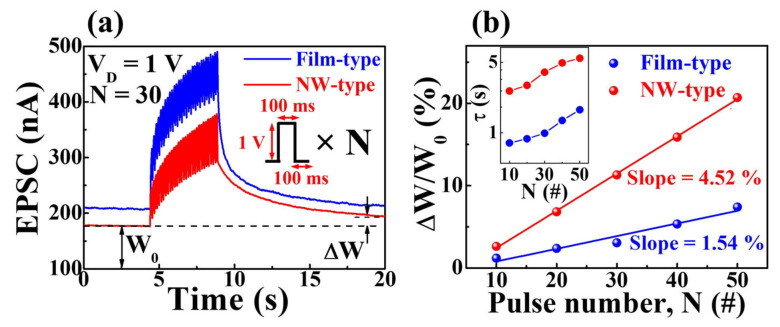
(**a**) EPSC response triggered by 30 successive gate pulses (N = 30) and (**b**) synaptic weight change ratio (ΔW/W_0_) as a function of the pulse number. The inset shows the relaxation time constant acquired by fitting the EPSC decay curve to an extended exponential function.

In the context of actual synapses, iterative rehearsals lead to the transition from short-term memory (STM) to long-term memory (LTM) [36,37]. Figure 4a shows the EPSC response elicited by 30 consecutive pulses (1 V, 100 ms), with the peak EPSC gradually increasing in both device configurations throughout the pulse. Moreover, the memory characteristics of the devices were quantitatively assessed by examining the synaptic weight changes following gate stimuli. These changes are denoted as ΔW/W_0_, where W_0_ and ΔW represent the initial drain current and the current change 10 s after the pulse sequence ends, respectively [18,38,39,40]. Figure 4b illustrates ΔW/W_0_ plotted against the pulse number, resulting in values of 1.2% and 2.6% for the film-type and NW-type devices at N = 10, respectively. As N increased to 50, these values increased to 7.4% and 20.7% with slopes equal to 1.54% and 4.52%, respectively, signifying a transition from STM to LTM. Notably, the NW-type device exhibits a larger ΔW/W_0_ value for the same pulse count. In electrolyte-based synapses, the onset of LTM is attributed to the electrochemical doping of protons into the IGZO channel, which is facilitated by significant gate energy (high-gate voltage or a substantial pulse count) [41]. For a deeper understanding, the inset of Figure 4b shows the relaxation time constant. Following repetitive gate stimulation, the decaying EPSC curve was well-fitted by the Kohlrausch stretched exponential function [41,42],

(2) $$\phi \left(t\right)=I_{0}\;\exp\left(-{\left(t/\tau \right)}^{\beta }\right)$$

where *ϕ*(t) represents the relaxation function, τ is the relaxation time constant, I_0_ is the prefactor, and β denotes the stretching exponent. τ tends to increase as a function of the pulse count, with the NW-type device exhibiting a higher relaxation time constant than its film-type counterpart. These findings suggest that NW-type channels adeptly emulate short- and long-term plasticity even with identical electrolyte and channel compositions. 

**Figure 5:**
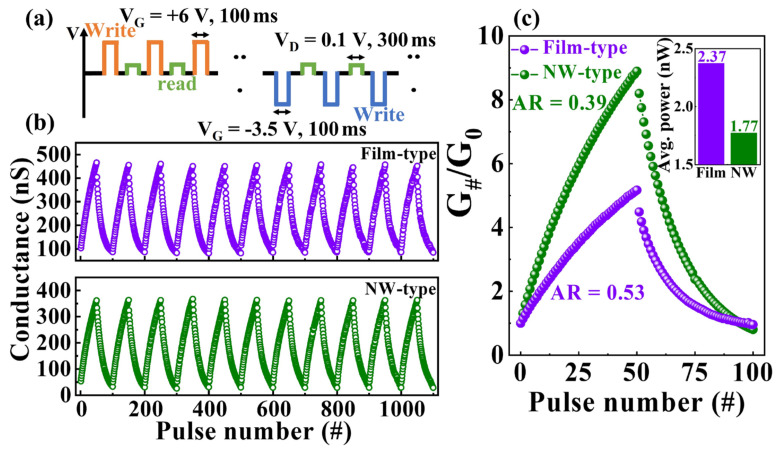
(**a**) Potentiation/depression pulse scheme and (**b**) gradual channel conductance modulation in film- and NW-type synaptic transistors. (**c**) Normalized conductance modulation curves (inset: average power consumption for the “read” process).

In biological synapses, weight updates occur gradually through external stimuli that induce potentiation or depression [43]. Figure 5 illustrates channel conductance modulation following the application of 50 potentiation (6 V, 100 ms) and depression (−3.5 V, 100 ms) pulses to the gates of film- and NW-type synaptic transistors at V_D_ = 0 V. Employing a high-gate voltage enables long-term plasticity changes with a single pulse. Figure 5a,b show the potentiation/depression pulse scheme and the conductance state measured by a drain read pulse (0.1 V, 300 ms), respectively. Both devices show stable conductance modulation over 11 cycles with a cycle-to-cycle variation (σ/μ) of <2%. The normalized potentiation and depression conductance values of the film- and NW-type devices are shown in Figure 5c. The conductance of each step (G_#_) was normalized to the initial conductance (G_0_) to determine the synaptic weight (G_#_/G_0_), which reflects synaptic strength. Nonlinearity analysis of normalized conductance reveals key properties—dynamic range (DR), asymmetry ratio (AR), and linearity—associated with learning and recognition accuracy. The DR, calculated as G_max_ divided by G_min_, signifies the conductance modulation. Higher DR values enhance the simulation performance and accuracy. DR values for the film- and NW-type synaptic transistors are 5.17 and 8.89, respectively, with the NW-type device exhibiting approximately 1.72 times higher values, thus indicating stronger modulation capability. The AR quantifies the asymmetry in potentiation and depression conductance alterations. As the AR values approached zero, the conductivity alteration became more symmetrical and resulted in enhanced learning accuracy. The AR was derived using the following equation [44],

(3) $$AR=\frac{MAX\left|G_{p}\left(n\right)-G_{d}\left(n\right)\right|}{G_{p}\left(30\right)-G_{d}\left(30\right)}\;for\;n=1\;to\;50$$

where G_p_(n) and G_d_(n) correspond to the nth potentiation and depression pulses, respectively. AR values of 0.53 and 0.39 were obtained for the film-type and NW-type devices, respectively, thus indicating closer proximity of the NW-type device to ideal and symmetrical conductance alterations. The linearity of the significant conductance in the recognition simulations was evaluated using the nonlinearity coefficient [45],

(4) G=Gmaxα−Gminα× w+Gminα1αGmin×(Gmax/Gmin)w      if α≠0,      if α=0.

where G_max_ and G_min_ are the maximum and minimum conductivity values, respectively, and w is an internal variable varying from zero to one. Additionally, α_p_ and α_d_ represent nonlinearity factors for potentiation and depression, respectively. For the NW-type device, α_p_ and α_d_ were extracted as 1.57 and −0.09, thus improving conductance alteration linearity compared with the film-type device (α_p_: 1.6, α_d_: −0.91). These parametric results suggest that the NW channels enhance the learning and recognition in ANNs. The inset in Figure 5c shows the average power consumption for a single weight-updating operation. In transistor-type artificial synapses, “write” and “read” powers relate to gate leakage and drain current, respectively. As the gate leakage is much lower than the drain current, power consumption during learning primarily occurs during “read” processes [46,47]. The average power consumptions for film- and NW-type synaptic transistors were 2.37 and 1.77 nW, respectively, thus indicating an approximate 25% power reduction from the NW channel. 

### 2.3. MNIST ANN Recognition Simulations

**Figure 6:**
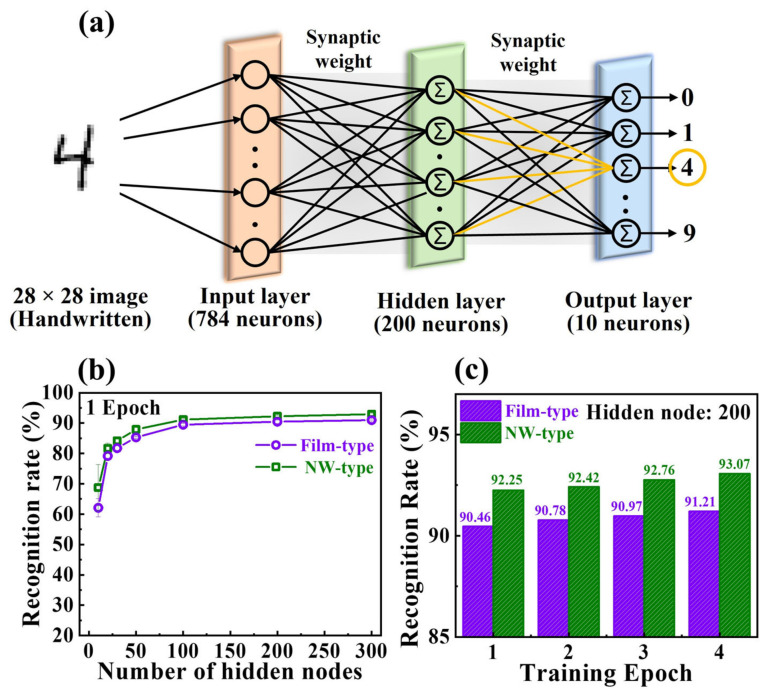
(**a**) Schematic outline of an artificial neural network model for MNIST recognition simulation with fully connected input, hidden, and output layers via synaptic weights. (**b**) Recognition rates corresponding to varying numbers of hidden nodes in epoch 1. (**c**) Recognition rates across multiple training epochs.

ANNs are computational frameworks that mimic complex functions, such as perception and recognition by interconnecting neurons, similar to the human brain. For neuromorphic systems, the ANNs must be constructed using synaptic devices at the hardware level [48]. Thus, by leveraging the analog potentiation/depression properties of the proposed film- and NW-type synaptic devices, we assessed the efficiency of ANN construction using training and recognition simulations on the MNIST digit database. The multilayer ANN model in Figure 6a illustrates the interconnected input, hidden, and output layers with synaptic weights. The input layer comprised 784 neurons representing the 28 × 28 MNIST dataset, whereas the output layer consisted of 10 neurons corresponding to the digits (0–9). The normalized conductance and acquired coefficients were integrated into the ANN model. This model was then used to evaluate neuromorphic computing using an MNIST learning test. The training was performed with approximately 60,000 simulated MNIST datasets, and the recognition accuracy was assessed by varying the number of hidden nodes (10–300). Figure 6b,c show the improved recognition accuracy with an increasing number of hidden nodes and training epochs, respectively. The NW-type device exhibited enhanced potentiation and depression characteristics that resulted in a higher recognition rate than that of the film-type device. The recognition rates for film- and NW-type EDL transistors (EDLTs) were 90.46% and 92.25% with 200 hidden nodes (in each of the two transistor types) and 91.21% and 93.07% after four epochs, respectively. Consequently, employing the NW channel structure in an EDLT is promising for high-performance artificial synapses. 

## 3. Conclusions

In this study, we introduced an innovative approach to enhance the transparency and flexibility of synaptic transistors using random-network IGZO NW channels in conjunction with a chitosan, hydrogel electrolyte. The investigation of these NW-type synaptic transistors compared with their film-type counterparts demonstrated substantial improvements in synaptic behavior and functionality. The incorporation of NW channels enabled the efficient modulation of channel conductance by inducing ionic polarization owing to their high surface-to-volume ratios. Consequently, the NW-type devices exhibited a rapid increase in the hysteresis window as the maximum V_G_ increased, thus indicating an enhanced synaptic response. Furthermore, the NW-type devices yield higher PPF indices which reach values up to 145%, thus indicating improved short-term plasticity. They also demonstrate larger ΔW/W_0_ values and higher relaxation time constants, signifying enhanced long-term synaptic capabilities. The exceptional linearity observed in the conductance modulation of NW-type devices coupled with a 25% reduction in power consumption, boosts their potential for practical applications. This is particularly relevant for constructing ANNs, as demonstrated by the higher pattern recognition accuracy achieved for the MNIST dataset using NW-type synaptic transistors in ANN simulations. These results enhance the feasibility of the use of NW-type synaptic transistors to develop low-power high-performance ANNs. The integration of NW channels into chitosan hydrogel electrolyte-based synaptic transistors holds great promise for the development of transparent and flexible electronics. This study contributes to the development of novel synaptic devices and provides insights into the realization of transparent and flexible neuromorphic systems with enhanced synaptic capabilities.

## 4. Materials and Method

### 4.1. Formation Process of IGZO NWs

**Figure 7:**
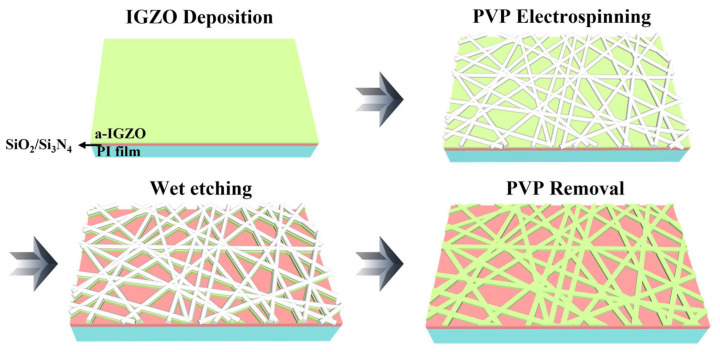
Fabrication process of indium-gallium-zinc oxide nanowires (IGZO NWs) utilizing a polyvinylpyrrolidone nanofiber (PVP NF) template.

To prepare flexible substrates, a polyimide (PI) layer (thickness = 6 μm) was spin-coated onto rigid glass. Subsequently, a protective SiN_x_/SiO_2_ layer (thickness = 100/100-nm) was deposited on the PI substrate using plasma-enhanced chemical vapor deposition to prevent potential chemical damage. An IGZO film (thickness = 20-nm) was sputtered onto a flexible and transparent PI substrate using an IGZO target (In_2_O_3_:Ga_2_O_3_:ZnO = 4:2:4.1 wt %) via radio frequency (RF) magnetron sputtering. To facilitate the subsequent NW fabrication, PVP nanofibers (NFs) were electrospun onto the IGZO film utilizing a PVP precursor solution and oven-baked for 10 min at 200 °C on a hot plate in an air environment. The PVP precursor solution was created by dissolving 200 mg of PVP powder in 3 mL of ethanol, then stirring at 800 RPM at room temperature for 4 h. This step was then used to create a wet-etch mask. The exposed regions of the IGZO channels were selectively etched using a 30:1 buffered oxide etchant (BOE), and the residual PVP NFs were removed via reactive ion etching (RIE) using O_2_ plasma ambient for 3 min at 50 W. This resulted in the successful transfer of the pattern from the electrospun PVP NF template to the IGZO film and the formation of a random network of IGZO NWs. Figure 7 illustrates the process by which the IGZO nanowires were formed by transferring the pattern from the PVP NF template. Notably, the electrospun PVP nanofibers consisted of only a few layers and exhibited a large open area. Owing to these substantial (open) areas, the exposed IGZO regions were selectively etched using a wet-etching process. The diameter of the PVP NFs used for the etching mask was approximately 500 nm, which was significantly larger than the IGZO layer thickness. This substantial size difference ensured minimal horizontal loss during the wet-etching process. 

### 4.2. Preparation of Chitosan Solution

A chitosan solution was prepared by dissolving a blend of 2% chitosan powder derived from shrimp shells in a 2% acetic acid solution with 10 mL of deionized water. The chitosan solution was fully dissolved using a magnetic stirrer at 800 revolutions per minute for 6 h at 50 °C. Subsequently, the solutions were filtered through a 5 μm pore size polytetrafluoroethylene syringe filter (Whatman, International Ltd., Maidstone, UK) to remove any impurities. 

### 4.3. Fabrication of Transparent and Flexible IGZO NW Synaptic Transistors

A flexible and transparent synaptic transistor was prepared featuring a random-NW network channel composed of IGZO. The device employed a chitosan-based hydrogel electrolytic gate dielectric. The fabrication process began with the formation of IGZO nanowires on a PI substrate. The active regions of the field-effect transistors were defined via photolithography and wet etching using a 30:1 BOE solution. For the source/drain (S/D) electrodes, a transparent indium-tin-oxide (ITO) film (thickness = 150 nm) was deposited via RF magnetron sputtering, and patterned using a lift-off process. Enhancing electrical characteristics and eliminating defects, the IGZO NW channel underwent thermal treatment at 250 °C for 30 min in nitrogen (N_2_) ambient conditions. Subsequently, the chitosan-based hydrogel electrolyte was spin-coated using a 2% chitosan organic solution. After air drying for 24 h, the hydrogel was oven-baked for 10 min at 130 °C. A Ta_2_O_5_ capping layer (thickness = 80 nm) was deposited by RF magnetron sputtering to protect the chemically vulnerable chitosan-based hydrogel electrolytes and enable photolithography [49]. Finally, an ITO, top-gate electrode (thickness = 150 nm) was formed on top of the Ta_2_O_5_ capping layer, and the S/D contact holes were opened using RIE with SF_6_ and O_2_ plasma. 

### 4.4. Characterizations

To minimize the impact of external optical and electrical noise, transparent and flexible IGZO film- and NW-type synaptic transistors were placed in an enclosed dark box on a dedicated probe station to ensure precise measurements. An Agilent 4156 B Precision Semiconductor Parameter Analyzer (Hewlett-Packard Co., Palo Alto, CA, USA) was used to measure the electrical and synaptic functions of the proposed IGZO film- and NW-type synaptic transistors. Synaptic modulation was assessed using electrical pulse stimulations generated by an Agilent 8110A pulse generator (Hewlett-Packard Co.). 

## Data Availability

The data presented in this study are openly available in article.
